# Supplementary material for: Entropic bonding of the type 1 pilus from experiment and simulation
Source: R Soc Open Sci. 2020 Apr 15;7(4):200183. doi: 10.1098/rsos.200183 (PMC7211842; doi:10.1098/rsos.200183)
Supplement: Supplementary Material document [file rsos200183supp1.pdf]

Royal Society Open Science

Supplementary Material for:
Entropic bonding of the type 1 pilus from experiment and simulation
Fabiano Corsetti, Alvaro Alonso-Caballero, Simon Poly, Raul Perez-Jimenez, Emilio Artacho

EXAMPLES OF REJECTED TRACES

Fig. S1 shows some examples of force-extension traces rejected by the automatic processing procedure.

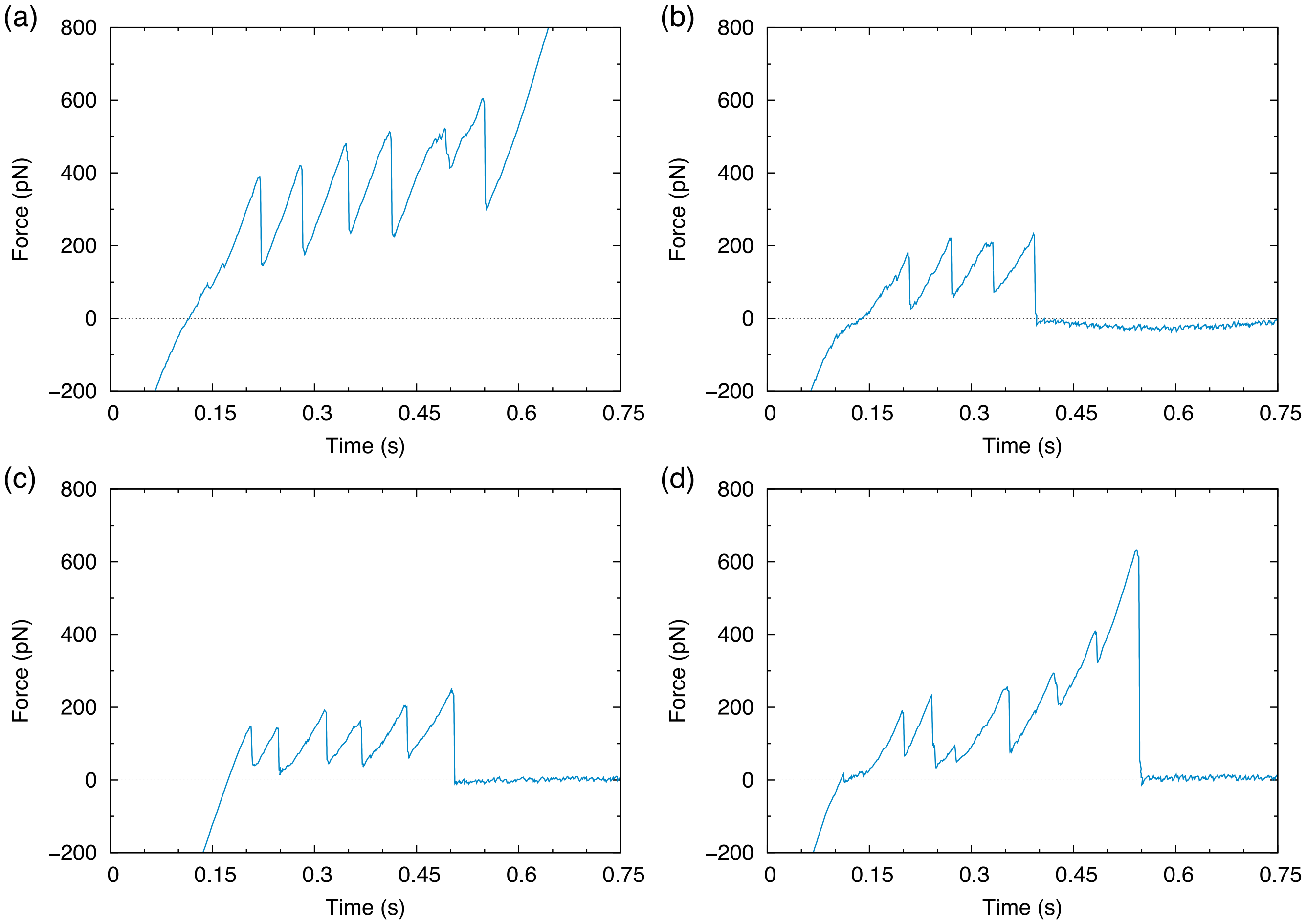

FIG. S1. **Examples of rejected experimental traces.** The reasons for rejection are: (a) no flat region at the end of the trace; (b) too few peaks; (c) fitted WLC curves not close enough to the real trace; (d) unreasonable values for the domain contour lengths.

WORM-LIKE CHAIN AND FREELY-JOINTED CHAIN MODELS

The worm-like chain (WLC) model used to fit the branches of the experimental traces is that of Bouchiat *et al.* [1], given by:

$$f = \left(\frac{k_\mathrm{B} T}{P} \right) \left[\frac{1}{4 \left(1 - x/s \right)^2} - \frac{1}{4} + \sum_{i=1}^{7} \alpha_i \left(\frac{x}{s} \right)^i \right], \tag{S1}$$

where f is the force, x is the end-to-end extension, s is the contour length, P is the persistence length, k_{B} is Boltzmann's constant, T is the temperature, and the coefficients of the polynomial expansion are $\alpha_1 = 1$, $\alpha_2 = -0.5164228$, $\alpha_3 = -2.737418$, $\alpha_4 = 16.07497$, $\alpha_5 = -38.87607$, $\alpha_6 = 39.49944$, $\alpha_7 = -14.17718$.

The freely-jointed chain (FJC) model used to fit the last part of the simulated traces is that of Smith *et al.* [2], given by:

$$x = s\left[\coth\frac{2Pf}{k_{\mathrm{B}}T} - \frac{k_{\mathrm{B}}T}{2Pf}\right]\left(1 + \frac{f}{K}\right), \qquad \text{(S2)}$$

where K is the elastic modulus.

[1] C. Bouchiat, M. D. Wang, J.-F. Allemand, T. Strick, S. M. Block, and V. Croquette, Biophys. J. **76**, 409 (1999).
[2] S. B. Smith, Y. Cui, and C. Bustamante, Science **271**, 795 (1996).
